# Hemodialysis without Systemic Anticoagulation: A Prospective Randomized Trial to Evaluate 3 Strategies in Patients at Risk of Bleeding

**DOI:** 10.1371/journal.pone.0097187

**Published:** 2014-05-13

**Authors:** Bruno Guéry, Corinne Alberti, Aude Servais, Elarbi Harrami, Lynda Bererhi, Brigitte Zins, Malik Touam, Dominique Joly

**Affiliations:** 1 Université Paris-Descartes, Faculté de Médecine, Assistance Publique-Hôpitaux de Paris, Service de Néphrologie, Hôpital Necker-Enfants Malades, Paris, France; 2 Université Denis Diderot, Assistance Publique-Hôpitaux de Paris, Hôpital Robert Debré, Unité d’Epidémiologie Clinique et Institut National de la Santé et de la Recherche Médicale CIE 5, Paris, France; 3 Institut National de la Santé et de la Recherche Médicale U845, Hôpital Necker-Enfants Malades, Paris, France; Maastricht University Medical Center, Netherlands

## Abstract

**Objective:**

In this clinical trial, we aimed to compare three means of performing chronic hemodialysis in patients with contra-indication to systemic heparinization.

**Methods:**

This open-label monocentric randomized « n-of-one » trial, conducted in a single tertiary care center, recruited chronic hemodialysis patients with a contra-indication to systemic heparinization for at least 3 consecutive sessions. All patients underwent hemodialysis with an AN69ST dialyzer, and were administered three alternative dialysis procedures in a random sequence: intermittent saline flushes, constant saline infusion, or pre-dialysis heparin coating of the membrane. The primary outcome was the need to interrupt the dialysis session because of clotting events due to either (i) a complete coagulation of the circuit; (ii) a partial coagulation of the circuit; (iii) a>50% rise over baseline in the venous pressure.

**Results:**

At the end of the inclusion period (May, 2007 to December, 2008), the number of patients to include (n = 75) was not reached: only 46 patients were included and underwent randomization. The study was terminated, and statistical analysis took into account 224 hemodialysis sessions performed in 44 patients with analyzable data. Heparin adsorption was associated with a significant reduction of the need to interrupt the dialysis session because of clotting events: odds ratio 0.3 (CI 95% 0.2 to 0.6; p<0.001, versus intermittent saline flushes). Heparin adsorption was also associated with higher odds for performing >3 h dialysis sessions and for having complete blood restitution. There were no significant effects of the dialysis procedure on weight loss, online ionic dialysance, and adverse events.

**Conclusion:**

Heparin-coated AN69ST dialysis membrane is a safe and effective method to avoid or delay per-dialytic clotting events in patients with contra-indication to systemic anticoagulation. However, results are not generalizable safely to patients with active bleeding, since weak heparinemia, not assessed in this study, may occur.

**Trial Registration:**

ClinicalTrials.gov NCT00473109.

## Introduction

Heparin, either unfractionated or fractionated, is commonly administered during hemodialysis sessions to prevent clotting events in the extracorporeal circuit [1]–[6]. This systemic anticoagulation is however contra-indicated in patients at high risk of bleeding. In these challenging cases, a variety of methods have been proposed^7^. Regional heparin/protamine [8], regional citrate/calcium [9]–[13] and prostacyclin [14], [15] are efficient but their use was limited by technical difficulties or clinical complications. Conversely, intermittent saline flushes (ISS) and constant saline infusion (CSI) of the circuit were described as simple and safe techniques to reduce clotting [7], [16], [17] and are widely used use in daily practice. Finally, the AN69ST membrane (polyacrylonitrile surface, modified by a polyethyleneimine layer) allows stable binding of unfractionated heparin [18]. Heparin coating of the AN69ST membrane produces a local antithrombogenic action which may help perform in some selected patients regular dialysis sessions without systemic heparinization [19], [20]. However, the benefits of this technique remained to be established in a comparative trial, especially in patients at risk of bleeding.

In this open-label monocentric randomized prospective study, patients at risk of bleeding that were included underwent hemodialysis without systemic heparinization; all dialysis sessions being performed with AN69ST membrane, we aimed to compare through an individual cross over “n of 1”-type trial the effects of intermittent saline flushes, constant saline infusion, and heparin-coating on clotting events.

## Materials and Methods

The protocol for this trial and supporting CONSORT checklist are available as supporting information; see Checklist S1 and Protocol S1.

### Patients

We planned to include adult patients on chronic hemodialysis who had a contra-indication to the use of systemic heparinization for at least three consecutive sessions, because of a high bleeding risk (active bleeding, recent surgery or organ biopsy, pericardic effusion), cholesterol emboli, or immunoallergy to heparin. We planned to exclude any patients with either an access blood flow <250 ml/mn, hemoglobin >13 g/dl, systolic blood pressure <80 mm Hg, or needing per-dialytic nutritional or blood transfusion support.

The protocol was approved by the local ethics committee (Comité Consultatif de Protection des Personnes en Recherche Biomédicale de l’Ile de France) and written informed consent was obtained from all the patients.

### Dialysis Procedures

Dialysis duration was 4 hours, three times per week. Vascular access was either a native arterio-venous fistula (bipuncture) or a double lumen tunneled catheter. Blood flow was wept between 250 and 300 ml/mn. Dialysate prescriptions were as follow: flow 500 ml/mn, sodium conductivity 14.4 mS/cm, bicarbonate conductivity 3.3 mS/cm, temperature 36°C. Ultrafiltration was performed as requested by patient’s weight gain and clinical status. All sessions were performed with Nephral 400 ST membranes (AN69ST, hollow-fiber 1.4 m2; Gambro-Hospal, Meyzieu, France).

The three specific procedures to avoid systemic heparinization tested in this study were:

Intermittent saline flushes: every 30 minutes, the blood arterial line was transiently occluded to allow the infusion and quick flush of 125 ml of 0.9% sodium chloride. Constant saline infusion: 0.9% sodium chloride was infused via the arterial line at a constant rate of 250 ml/h during the whole session. Heparin coating was performed according to the manufacturer’s recommendations: the AN69ST membrane was primed with 2 liters 0.9% saline containing 10 000 IU of heparin.

### Outcomes and Measurements

The primary outcome was defined as the need to interrupt the dialysis session because of clotting events accounting for either (i) a complete coagulation of the circuit; (ii) a partial coagulation of the circuit; (iii) a>50% rise over baseline in the venous pressure. For each dialysis session, we recorded patient’s blood pressure and heart rate, initial, dry and final weight, on-line ionic dialysance values (every 30 mn), eventual clinical symptoms and dialysis duration. We also recorded the medical history, treatments. Any adverse events was also recorded; the observation interval was not limited to the duration of the dialysis session, but was extended to the beginning of next dialysis session.

### Study Design, Location and Sample Size Calculations

This study was performed at a single tertiary care center (Necker Hospital, Paris). Patients fulfilling all inclusion and exclusion criteria were included in this « N of one » trial in which the three alternative dialysis procedures tested were administered for each patient in a random sequence. The dialysis procedures were used in an open label fashion. The random sequence was generated by computer in an independent center and sent to us by fax. When more than 3 sessions without systemic heparinization had to be performed, order of the three additional sequences was randomly changed. Patients were withdrawn from the study when bleeding risk has disappeared, or after completion of 36 sessions, or if three consecutive complete circuit clotting had occurred with the same specific procedure.

Sample size calculation was based on MacNemar test comparing matched groups for a binary test. Based on our previous unpublished retrospective experience, we hypothesized that the rates of primary outcomes would be 30% with intermittent saline flushes (ISF, standard technique), 20% with constant saline infusion (CSI), and 10% with heparin adsorption (HA). Based on this, with a risk alpha of 5% and a power of 80%, we planned to include 65 pairs of sessions ISF and CSI and 294 pairs of ISF and HA. Thus, 294 triplets of sessions were retained and were inflated to 350 to take into account the clustering of patients (our estimation of a cluster size of 6 and of an intraclass correlation of 0.04 resulted in an inflation factor coefficient of 1.19). Consequently, 75 patients had to be recruited in 18 months time. Based on this calculation, it was decided that the study would be monocentric.

### Statistics

Qualitative variables were described as number (percentages) and quantitative variables as median (1st and 3rd quartiles). We built a two levels hierarchical logistic mixed model for qualitative outcomes as interruption of dialysis session and linear mixed model for quantitative outcomes. Level one represented the dialysis sessions as fixed effect and level two the patient as random effect. Results are expressed in Odd Ratio and regression coefficient with their 95% confidence interval (with intermittent saline flushes as the reference method). All tests were bilateral and statistical significance was stated to 5%. Data management was performed with SAS 9.2 (SAS Institute, Cary, NC) and hierarchical models building used MLwiN v2.0.

## Results

### Patients and Treatment Allocations

At the end of the defined inclusion period (18 months, between May, 2007 and December, 2008), the initially planned number of patients to include (n = 75) had not been reached. During this period, only 48 successive patients on chronic hemodialysis fulfilling all inclusion and exclusion criteria were considered for potential enrolment. One patient declined to be enrolled. Another patient was excluded because his consent form was lost. A total of 46 patients agreed, were included in the study, and underwent randomization. Two additional patients withdrew their consent. The investigators and the sponsor decided to terminate the study and to perform the statistical analysis.

**Figure 1:**
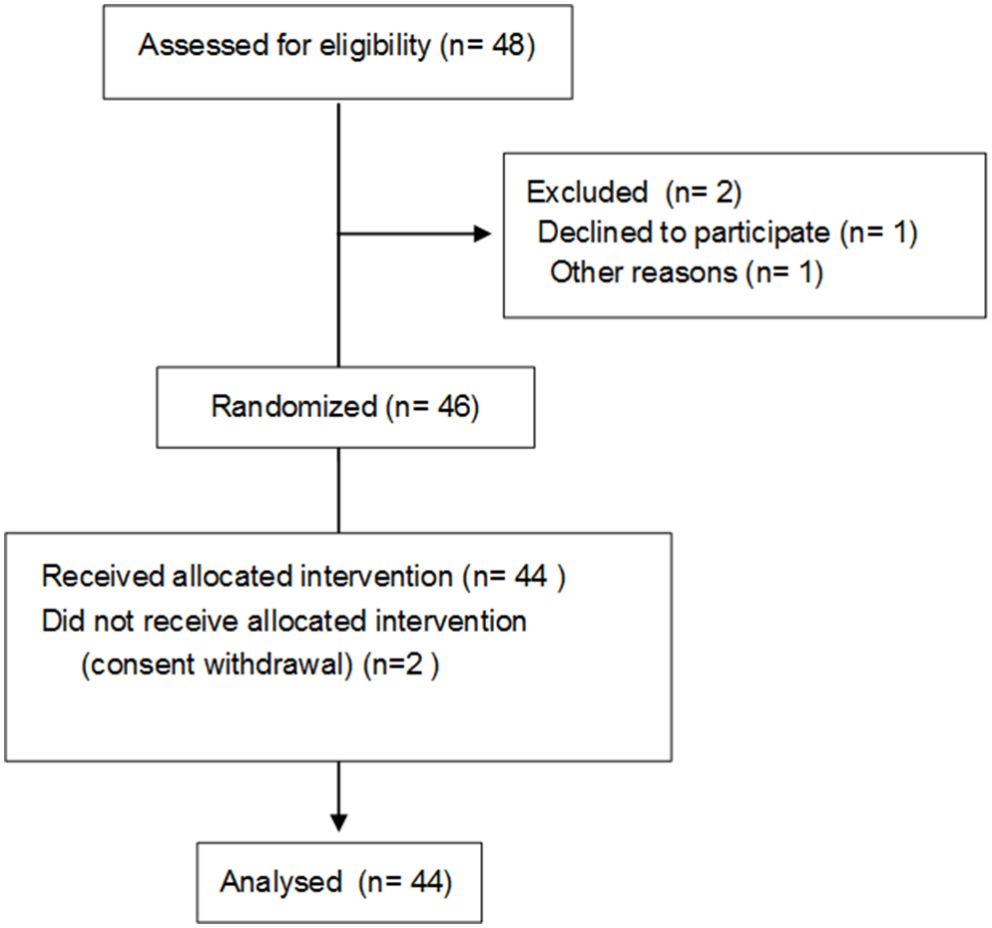
Flow Diagram.

Patient’s flow diagram, relevant characteristics and reasons for not performing systemic heparinization are indicated in figure 1 and table 1. This rather unselected group of patients was characterized by a relatively young median age, a reduced dialysis vintage, and a high proportion of patients undergoing urologic surgery (including nephrectomy for 5 patients with autosomal dominant polycystic kidney disease).

**Table 1:** Characteristics of the 44 patients at inclusion.

Variables	median (Q1; Q3) or n (%)
Age (years)	55.3 (42.4; 72.8)
Male gender	22 (48%)
Weight (kg)	63.0 (54.5; 70.5)
Hemoglobin (g/dl)	11.1 (9.2; 12.0)
Diabetes	12 (26%)
Dialysis vintage <12 months	39 (84%)
Native arterio-venous fistula	20 (43%)
Central tunnelized catheters	26 (56%)
Reasons for not using heparin	Recent surgery, n = 29
	Recent organ biopsy/embolization, n = 8
	Current bleeding, n = 6
	Hematologic disorders/thrombopenia, n = 3
	Pericardic effusion, n = 1

Patients underwent a median of 3 sessions (range: 2–36). A total of 224 sessions was performed: 74 with ISS, 74 with CSI, and 76 with HA. Overall, the order in the random sequence was comparable for the three dialysis techniques.

### Clotting Events

Clotting events leading to premature termination of the dialysis session were observed in 51.4%, 55.4% and 23.7% of dialysis sessions and in 67.4%, 73.8% and 37.2% of patients using ISS, CSI and HA, respectively. Heparin adsorption technique conferred lower odds for premature termination of the dialysis session (OR = 0.3; 95% Confidence Interval, CI (0.2–0.6); p<0.001). HA was also associated with lower rates of, and fewer patients with, complete clotting of the circuit, partial clotting, and with >50% increase in venous pressure (table 2). Finally, complete blood restitution was more frequently reported after HA than with ISS or CSI (respectively 75%, 48.7% and 47.3%, p = 0.001).

**Table 2:** Clotting events.

Variables		Intermittent Saline flushes	Continuous Saline infusion	Heparin adsorption
Interrupted sessions	n sessions/n patients	74/43	74/42	76/43
	n sessions (%)	38/74 (51%)	41/74 (55%)	18/76 (24%)
	n patients (%)	29/43 (67%)	31/42 (74%)	16/43 (37%)
	OR (95%CI), p	1.0	1.2 (0.6–2.3), p = 0.6	0.3 (0.2–0.6), p = 0.0005
Complete clotting	n sessions (%)	31/74 (42%)	32/74 (43%)	14/76 (18%)
	n patients (%)	25/43 (58%)	24/42 (57%)	13/43 (30%)
	OR (95%CI), p	1.0	1.1 (0.6–2.1), p = 0.8	0.3 (0.2–0.6), p = 0.001
Partial clotting	n sessions (%)	14/74 (19%)	13/74 (18%)	16/76 (21%)
	n patients (%)	11/43 (26%)	10/42 (24%)	14/43 (33%)
	OR (95%CI), p	1.0	0.9 (0.4–2.1), p = 0.9	1.2 (0.5–2.6), p = 0.7
Venous pressure rise >50%	n sessions (%)*	36/73 (49%)	37/74 (50%)	19/76 (25%)
	n patients (%)*	27/42 (64%)	28/42 (67%)	17/43 (40%)
	OR (95%CI), p	1.0	1.0 (0.5–2.0), p = 0.9	0.3 (0.2–0.7), p = 0.002
Incomplete blood restitution	n sessions (%)	38/74 (51.3%)	39/74 (52.7%)	19/76 (25.0%)
	OR (95%CI), p	1.0	1.1 (0.6–2.0), p = 0.9	0.3 (0.2–0.6), p = 0.001

OR: Odd Ratio, CI: Confidence Interval. OR were computed using a two levels hierarchical logistic mixed model.

*data were missing for one patient.

### Dialysis Duration, Performance and Tolerance

Dialysis sessions performed with the heparin adsorption technique were longer than with other techniques, with a significantly higher probability of lasting more than 3 hours (table 3). Total weight loss was not statistically higher with the heparin adsorption technique. Instantaneous urea nitrogen blood clearances measured after 3 hours of dialysis were not statistically different with the three techniques (table 3). Bleeding of any cause was recorded after 18 of the 224 dialysis sessions (4.5%) performed in this study (the observation period included the dialysis session and the time frame until the beginning of the next dialysis session). Bleeding episodes were never judged as severe, nor required red blood cells transfusion, and were not statistically more frequent after sessions performed with the heparin adsorption technique (p = 0.69 by Fisher’s exact test). All dialysis sessions associated with a reported bleeding episode were performed in the 6 patients included in this study because of an active bleeding (see table 1). We did not observe any new or unexpected bleeding during the study.

**Table 3:** Dialysis efficiency and tolerance.

Variables		Intermittent Saline flushes	Continuous Saline infusion	Heparin adsorption
Dialysis session length (hours)	median (Q1; Q3)	3.5 (2.5; 4.0)	3.6 (2.5; 4.0)	4.0 (3.9; 4.0)
Dialysis >3 hours	n (%)	42/74 (61%)	47/74 (64%)	67/76 (88%)
	OR (95%CI), p	1.0 (ref.)	1.1 (0.6–2.1), 0.8	4.7 (2.2–10.4), <0.001
Weight loss (kgs)**	median (Q1; Q3)	2.0 (1.3; 2.8)	1.8 (1.2; 2.4)	2.4 (1.6; 2.8)
	RC (95%CI), p	0	−0.2 (−0.6; 0.2), 0.3	0.2 (−0.2; 0.6), 0.3
Ionic dialysance (mL/mn)*	median (Q1; Q3)	173 (162; 184)	170 (162; 182)	170 (161; 179)
	RC (95%CI), p	0	−4.3 (−11.6; 2.62), 0.1	−4.9 (−12.0; 2.3), 0.09
Bleeding after dialysis session	n sessions (%)	3/74 (4%)***	5/74 (7%)***	6/76 (8%)***
Headache	n sessions (%)	0/74 (0%)	0/74 (0%)	2/76 (3%)
	n patients (%)	0/43 (0%)	0/42 (0%)	1/43 (2%)
Cramps	n sessions (%)	1/74 (1%)	0/74 (0%)	2/76 (3%)
	n patients (%)	1/43 (2%)	0/42 (0%)	2/43 (5%)
Symptomatic hypotension	n sessions (%)	0/74 (0%)	1/74 (1%)	1/76 (1%)
	n patients (%)	0/43 (0%)	1/42 (2%)	1/43 (2%)
Other symptoms	n sessions (%)	2/74 (3%)	1/74 (1%)	3/76 (4%)
	n patients (%)	2/43 (5%)	1/42 (2%)	3/43 (7%)

CI: confidence interval. OR: Odd Ratio, RC: Regression coefficient, ref: reference OR were computed using a two levels hierarchical logistic mixed model and regression coefficient with a two levels hierarchical linear mixed mode.

*missing data for 48 sessions;

**missing data for 23 sessions.

***p = 0.69 by Fisher’s exact test.

Perdialytic clinical events including headache, cramps, and hypotension were infrequent in this study (listed table 3). The proportion of sessions and the proportion of patients experiencing these events were similar between the three dialysis techniques.

## Discussion

To compare the three dialysis modalities, we performed a n-of-one trial. A n-of-1 trial is a crossover trial conducted in an individual patient. Its results may indicate the best treatment at the individual level. Combining the results of a series of similar n-of-1 trials conducted in different patients may indicate the best treatment at the collective level. The crossover design of n-of-1 trials is particularly appropriate to study of clotting events during hemodialysis sessions: patients have a chronic condition requiring iterative therapeutic interventions; the treatments applied have short-term effects; and finally, measurements in each period are independent [21].

The budget allocated to this study did not allow us to prolong the inclusion period, at the end of which the planned number of patients (n = 75) had not been reached. The number of patients had been calculated based on the assumption that heparin adsorption would reduce the percentage of clotting system by 30%. The decision was made to terminate the study and to perform the statistical analysis, taking the risk of reporting an underpowered study. However, our data clearly indicate that, compared to intermittent saline flushes and continuous saline infusion, heparin-coating significantly reduced the rate of clotting events necessitating the premature termination of the dialysis session.

Clotting in the extracorporeal circuit depends on the anticoagulation procedure, the thrombogenicity of the different circuit components, the patient’s thrombophilic potential, and the fluid dynamics. The use of the same membrane in the three modalities strongly suggest that the differences reported in our study are mainly due to differences in the membrane thrombogenicity, the latter being reduced by heparin adsorption. The properties and specificities of the AN69 ST membrane have been underscored by many reports [18], [19], [22]; it should however be stressed here that heparin priming has also been reported with other synthetic membranes, including FX100 polysulphone [23].

Even with the heparin adsorption technique, clotting events occurred quite frequently. However, the rate of complete clotting in our study (18% of sessions) compares favorably to the 38% reported by Evenopol et al. in a study including also patients at risk of bleeding [24]. Part of this difference may be team-dependent; technical experience of the nurses is indeed critical to handle properly heparin-coated AN69 ST membranes, including careful deaeration of the dialyzer and hourly lowering of the blood column in the venous drip chamber, as recommended by the manufacturer. Patient’s risk for thrombosis was also probably lower in our study, due to selective inclusion criteria, which required vascular access flow >250 ml/mn and hemoglobin <13 g/dl. Of note, the incidence of massive clotting with the HA technique could be as low as 1/66 sessions in a different population, made of carefully selected stable hemodialysis patients with no specific risk of bleeding [19].

Two observations may help reduce the rate of clotting events in the future. First, almost all clots observed with heparin-coated AN69ST technique were initially formed in parts of the circuit not coated with heparin (mainly the venous bubble trap). Some authors have reported encouraging results with covalent coating of the whole extracorporeal circuit with low molecular weight heparin [25]. Second, the vast majority of clots observed with heparin-coated AN69ST formed at the very end of the dialysis session, suggesting that a slight reduction of the session length could have dramatically reduced the rate of coagulation events.

In this study, heparin adsorption did not improve membrane permeability to small molecular weight plasma molecules, as measured by online ionic conductance throughout sessions. Other groups have reported that during heparin-free dialysis, small solutes removal could be maintained despite moderate dialyzer clotting, to a possible increase of blood flow through the unclotted fibers [19], [26].

Heparin partial desorption from the membrane and incomplete removal of the priming solution before patient’s connection to the extracorporeal circuit may occur with the HA protocol, with subsequent systemic anticoagulation due to circulating heparin. Coagulation parameters were not assessed in the present study. However, antiXa activity measurements reported by Lavaud et coll. after 3 hours of dialysis were weakly positive, all values being <0.1 UI/ml [19]. Another study using heparin-coated membranes reported a weak and transient increase in activated partial thromboplastin time [22]. Even if the systemic exposure to heparin is transient and minimal, we consider that heparin-coated membranes should be strictly avoided in patients with heparin-induced allergy. The heparin adsorption protocol should probably also be avoided in patients who suffer from major active bleeding. Only 6 patients with active bleeding were included in our study. Besides patients with active bleeding, our results cannot safely be generalized to patients with acute renal failure, to patients in intensive care units, and to patients with different baseline characteristics, i.e very elderly patients and patients with high dialysis vintage.

In our experience, both ISS and CSI were associated with a very high rate of clotting events, confirming that their efficiency should be questioned [27]. By comparison, the heparin-coated AN69ST membrane technique appeared as a safe and effective method for providing hemodialysis treatments in most (if not all) patients with contra-indication to systemic anticoagulation. Despite its increased cost, this technique has become the preferred anticoagulation strategy in patients at risk of bleeding in our center. However, hemodialysis centers with access to both heparin-coated membranes and sodium citrate regional anticoagulation should be aware that the latter technique may provide an even superior anticoagulation efficacy, at the expense of a higher complexity [24].

## Supporting Information

Checklist S1 **CONSORT Checklist.**
(DOC)

Protocol S1 **Trial Protocol.**
(DOCX)
