# Supplementary material for: Hemodialysis without Systemic Anticoagulation: A Prospective Randomized Trial to Evaluate 3 Strategies in Patients at Risk of Bleeding
Source: PLoS One. 2014 May 13;9(5):e97187. doi: 10.1371/journal.pone.0097187 (PMC4019548; doi:10.1371/journal.pone.0097187)
Supplement: Protocol S1 — Trial Protocol. (DOCX) [file pone.0097187.s002.docx]

Direction de la Politique Médicale

Délégation Régionale à la Recherche Clinique

Hôpital Saint Louis

75010 PARIS

# HDSH trial

**Chronic hemodialysis without systemic heparinization**

## PRINCIPAL INVESTIGATOR

Dr Dominique Joly

Service de néphrologie

Hôpital Necker, 149 rue de Sèvres

75015 PARIS

Tel : 01 44 49 54 56 Fax : 01 44 49 54 53

###### Méthodologist

Dr Corinne ALBERTI

Unité d’Epidémiologie Clinique, Inserm CIE 5

Hôpital Robert Debré

75019 PARIS

Tel : 01 40 03 23 45 Fax : 01 40 03 24 85

**PROMOTOR**

AP-HP represented by the DRRC

Délégation Régionale à la Recherche Clinique, Hôpital Saint-Louis

1 avenue Claude Vellefaux, 75010 Paris

Tél : 01 44 84 17 88 Fax : 01 44 84 17 99

**CONTACTS**

**Investigator** :

Dr Dominique Joly

Service de Néphrologie

Hôpital Necker, 149 rue de Sèvres

75015 PARIS

Tel : 01 44 49 54 56 Fax : 01 44 49 54 53

**Co-investigators :**

Dr Bruno Guéry

Dr Lynda Bererhi

Dr Brigitte Zins

Service de Néphrologie

Hôpital Necker, 149 rue de Sèvres

75015 PARIS

Tel : 01 44 49 54 56 Fax : 01 44 49 54 53

**Methodology and Statistical analysis :**

Dr C. ALBERTI

Unité d’Epidémiologie Clinique, INSERM CIE 5

Hôpital Robert Debré

**Project Leader**

Christophe Aucan

Project Leader

Département de la Recherche Clinique et du Développement

(DRRC ILE-DE-FRANCE)

Tel : 01 44 84 17 23 Fax : 01 44 84 17 88

**Clinical Research Unit**

Unité de Recherche Clinique Paris Nord

Pr Philippe Ravaud

Tél : 01 40 25 62 51

# CONTENTS

### 1 Summary 4

2 **Introduction 5**

**3 Objectives 7**

**4 Experimental plan 8**

**5 Design and conduct of research 9**

**6 Procedures evaluated 10**

**7 Endpoints 10**

**8 Statistical data management 11**

**9 Safety issues monitoring and management 13**

**10 Legal and ethics aspects 14**

**Bibliography 18**

**Chronic hemodialysis without systemic heparinization**

**1- Summary**

Conventional hemodialysis requires systemic transient anticoagulation due to the activation of coagulation by contact between the patient's blood and the extracorporeal circuit. This anticoagulation, which uses heparin - fractionated or not – is however contra-indicated in patients with a high risk of bleeding. In this relatively common situation ( 10% of cases ), many procedures help perform hemodialysis without systemic anticoagulation, but no prospective study compare them. The reference method (" rinses " of the dialysis circuit with saline) is logistically cumbersome and subject to a relatively high failure rate. Two methods are emerging increasing use of : the continuous dilution of the dialysis circuit, and the exclusive anticoagulation the dialysis membrane by prior adsorption of heparin on a membrane having a treated non electronegative surface (NEPHRAL ST 400).

The single-center prospective randomized open controlled trial aims to compare " rinses ", " dilution " and " heparin adsorbed " in patients with a high risk of bleeding. The criteria will be clotting of the dialysis circuit (primary endpoint) and the efficacy and safety of dialysis ( secondary endpoints). This comparison will be made on the basis of statistics of randomized cross-over in unique individuals (" N- of- 1 trial ") series. We plan the inclusion of 78 different patients and implementation of 1050 dialysis without systemic heparinization.

We hope to show that, compared to " rinse " the " dilution " the " heparin adsorbed " technique reduces the frequency of coagulation circuit 30 and 50 %, respectively (primary endpoint) . We also hope to demonstrate that heparin adsorption allows ( i) better preservation of the porosity of the dialysis membranes, maintaining throughout the session a satisfactory removal of uremic waste, (ii) by limiting the use of fluids, helps to reach more often at the end of the session the dry weight of the patient and / or are to better clinically tolerate the dialysis sessions (secondary endpoints).

**2- Introduction**

Patients with end stage renal disease undergo 3 dialysis sessions per week (4 hours per session). Part of their blood (300 ml/minute) is derived by an extraorporeal circuit, and is dialysed in contact with an artificial membrane.

Despite numerous technological, a significant bioincompatibility persists between the blood and the dialysis membrane, which is responsible for complement and coagulation activation (Bartels, Schoorl et al. 2000) (Martin-Malo, Velasco et al. 1993). To avoid coagulation of the dialysis circuit, low molecular weight heparin (LMWH) are used (single bolus when the dialysis session starts) (Camici, Evangelisti et al. 1997). LMWH have supplanted continuous heparin infusions because their use is simpler and their efficiency is comparable (Nurmohamed, ten Cate et al. 1991; Weitz 1997). LMWH provide an excellent extraorporeal circuit anticoagulation, but also induce an efficient systemic anticoagulation of the patient that lasts about 12 heures (Bouffard, Delafosse et al. 1989). Their use is thus at risk, and may be contra-indicated (temporarily or definitively) in case of high bleeding risk.

In such circumstances, several dialysis techniques may avoid systemic anticoagulation (Caruana, Raja et al. 1987; Canivet, Wuillai et al. 2003):

- **Regional anticoagulation**
  - heparin/protamin : heparin is infused continuously in the arterial line of the circuit, while protamin, also infused continuously, neutralises it in the veinous line. Precise adjustment allowing a sufficient anticoagulation in the circuit without systemic anticoagulation is tricky to obtain : 35% of circuit clotting and 25 % of bleeding complications during the dialysis sessions have been reported (Hampers, Balufox et al. 1966) (Lindholm and Murray 1964) (Swartz and Port 1979) (Swartz 1981). This technique is no longer in use.
  - citrate/calcium : anticoagulation results form calcium chelation in the extracorporeal circuit by the sodium citrate continuously infused in the arterial line; the dialysate contains not calcium, and no magnesium (that may block the effects of citrate), but those are reinjected by continuous infusion at the end of the circuit (Pinnick, Wiegmann et al. 1983). This technique must be adapted to patient’s clotting tests and calcemia. Its efficiency is demonstrated, but it is complex, requires a specific dialysate, confers risks of dyscalcemia, metabolic alcalosis, hypernatremia, hyperammoniemia, and is frequently poorly tolerated (nausea, paresthesia, cramps, hypotension) in case of citrate overload (Flanigan, Von Brecht et al. 1987; Kelleher and Schulman 1987; Janssen, Huijgens et al. 1993; Apsner, Buchmayer et al. 2001).
  - AN 69 ST: recently described, this dialysis membrane made of polyacrylonitrile with modified surface (relative disparition of electronegative charges) can adsorb heparin. It is therefore possible to rince the circuit with heparinized saline, and to start the dialysis sessions without adding any anticoagulant, the heparin bound to the membrane playing a local role. Partial or massive clotting were observed in only 10 % of the sessions, in various studies (Delawari, Renaux et al. 2001; Lavaud, Canivet et al. 2002; Lavaud, Canivet et al. 2003) (Tielemans, Neyens et al. 2001).
- **Hémodialysis without anticoagulation**.
  - "discontinuous rinses" iterative rinses dialysis circuit with isotonic saline (from 50 to 300 ml every 15-30 min), have reduced the frequency of coagulation of the circuit to 30%. Some teams added to rinse prophylactic change of the membrane and dialysis circuit during the session (Preuschof, Keller et al. 1988). It is currently the gold standard hemodialysis when systemic anticoagulation is contra-indicated. Its implementation, however, requires substantial nursing work, and involves a nurse per patient (versus a nurse for four patients) throughout the session.
  - "predilution". Many teams use in practice a technique in which a flushing of the dialysis circuit is continuous (infusion of 4 cc of saline per minute in the arterial line). This technique is simple to implement, has never been validated as renal replacement without anticoagulation technique. The risk of clotting system is anticipated to 20%.

***Justification of research***

The main risks associated with the absence of systemic anticoagulation during a dialysis session are:

o complete extracorporeal circuit clotting, causing a blood loss of 300 ml. Iterative spoliation are often responsible for anemia requiring transfusions.

o reduction in the efficiency of dialysis, either by shortening the duration of the session (coagulation) or by micro-sealing of the dialysis membrane (clots invisible to the naked eye). The decrease in efficiency results in insufficient removal of uremic toxins (eg urea) and incomplete salt depletion (dry weight of the patient not reached).

Currently in most dialysis centers, including ours, three hemodialysis techniques without systemic anticoagulation mentioned above are used:

o " rinses" of the circuit with normal saline.

o " predilution " by continuous flushing of the circuit with isotonic saline.

o the use of AN69 ST membrane with adsorbed heparin.

There is at present no studies comparing different techniques of dialysis without systemic anticoagulation. In practice, the choice of which technique to use is primarily based on any rational standard and is left open to the discretion of the physician. It is very common to see in later sessions for the same patient, a strange alternation between different hemodialysis without anticoagulation techniques. These changes sometimes take prescriptions habits or preferences of physicians and dialysis nurses, the more often they are the waning of an accident clotting system. For example, a patient treated with " discontinuous rinses " at its nth session may, in case of clotting, have at the next dialysis session a " dilution " or an "adsorbed heparin" protocol.

*Expected Results and prospects*

In this study we want to compare, on the basis of test series randomized cross-over in unique individuals, three techniques in patients who have a contra-indication to systemic anticoagulation and / or a high risk of bleeding.

Based on retrospective observations conducted in our center last year, we hope to show that, compared to "rinses" (as a reference), the techniques of "dilution "and "adsorbed heparin " reduce the frequency of coagulation circuit 30 and 50%, respectively (primary endpoint).

We also hope to demonstrate that the technique of "adsorbed heparin" ( i) better preserves the porosity of the dialysis membranes, maintains throughout the session a satisfactory removal of uremic waste ( Romao , Fadil et al. 1997), (ii) avoids the intake and ultrafiltration liquid rinse or dilution, helps to reach more often at the end of the session the dry weight of the patient and / or are better tolerated clinically (secondary endpoints).

Dilution and adsorbed heparin are used by a small but growing number of nephrologists during hemodialysis sessions without LMWH. The results of the first prospective trial in the field would rigorously validate their effectiveness, and promote their recognition and distribution within the nephrology community. Implementation throughout the course of these two dialysis techniques without systemic heparinization would theoretically have several advantages (our study did not seek to assess), such as reduced transfusion requirements or erythropoietin, or the reduction of nursing work .

In daily practice, the results of this study will be of great use for the whole community : the superiority of the dilution over the rinse could be generalized to all types of dialysis membranes, while the technique of adsorbed heparin would be exploited by the centers using AN69 ST membrane, which cost is quite high.

**3- Objectives**

**Hypotheses tested**

The technique of " discontinuous rinsing " is currently the gold standard but has a significant cost (equipment and nursing time). We would like to validate the technique of "dilution" under the assumption that it will reduce the percentage of clotting system by 30% compared to the " discontinuous rinses."

The " adsorbed heparin technique " using the AN69 ST membrane is a technological breakthrough and we hope to show that the percentage of clotting decreases by 50% compared to the "discontinuous rinses".

**Primary objective**

The objective of this research project is to compare the dialysis techniques (discontinuous rinses, dilution, adsorbed heparin) in patients with temporary or definitive contra-indication to heparin during their dialysis session, on the risk of complete coagulation of the dialysis circuit.

**Secondary objectives**

The secondary objectives are to compare the dialysis techniques (discontinuous rinses, dilution, adsorbed heparin) in patients with temporary or definitive contra-indication to heparin in terms of efficacy and safety the dialysis session.

**4- Experimental plan**

***Choice of the experimental plan and justification***

It is a single-center, randomized, open-type N-of-1 trial (cross-over test series randomized in unique individuals). This is an open study since the techniques cannot be blinded ; however the endpoint chosen is objective (clotting or non-clotting of the dialysis circuit), and not amenable to interpretation. Moreover, the patient is not necessary blinded because this should not influence the response. The choice of N of 1 trial is justified because it is important to choose the best technique according to the patient, and the experimental design can provide an individual response and assess the repeatability of the response by the same technique in the same patient. This experimental design is made possible due to the completion of three dialysis sessions per week chronically ineffective a dialysis session to the next session with a rapid assessment (the same day as the dialysis session) of the test main judgment and fact will be selected patients with contra-indication for LMWH for at least 3 consecutive dialysis sessions without heparin. In addition, this experimental design allows techniques comparison across treatment groups.

The unit of randomization will be patient and will be made according to the following scheme:

- A = discontinuous rinses, B = dilution and C = adsorbed heparin
- The 6 possibles sequences of treatments are:
  - ABC
  - ACB
  - BAC
  - BCA
  - CAB
  - CBA

The patient will be randomized into one of six arms and make sure to have a number of patients included multiple of 6 (see calculation of the number of subjects required). Randomization will be by random blocks of varying size. The order of the types of sessions vary all three triplets for the same patient if the dialysis without heparin must be repeated beyond the first triplets. As a patient may contribute to several sequences, the patient will be considered as a cluster randomization.

***Selection criteria for patients***

Inclusion criteria:

- Patients (male or female) older than 18 years

- Hemodialysis due to chronic end stage renal disease

- Contraindications to the use of LMWH in hemodialysis, planned for at least three consecutive sessions because of:

o high risk of bleeding

• active bleeding, whatever the origin

• recent surgery or puncture body (<15 days)

• curative treatment with unfractionated heparin or anti-vitamin K.

o uremic pericarditis

o disease cholesterol emboli

o thrombocytopenia heparin

- Informed Consent signed

**Exclusion criteria:**

- Acute renal failure

- Nonfunctional vascular access (blood flow rate below 250 ml /mn)

- Need for perdialytic transfusion of blood products

- Need for perdialytic parenteral nutrition

- Severe arterial hypotension (systolic blood pressure less than 80 mmHg)

- Hemoglobin greater than 13 g/dl

Rationale: risk factors for clotting of the extracorporeal dialysis circuit are the duration of the session, a blood flow of less than 250 ml / min, performing of a blood transfusion or parenteral nutrition during the dialysis session, and hemoglobin > 13 g / dl (Keller, Seemann et al. 1990) (Schultze, Hollmann et al. 1992) (Sepulveda, Davis et al. 1997) (Sperschneider, Deppisch et al. 1997).

***Mode of recruitment***

Monocentric study: patients treated by chronic hemodialysis at the Necker Hospital will be included. Given the number of sessions performed annually at our center (about 7500), the frequency of dialysis prescriptions without systemic anticoagulation (about 10% of cases) representing fifty patients a year to meet the inclusion criteria, we expected inclusion of a period of 18 months would be necessary.

***Expected number of people and justification***

In this study, 78 patients will be included. See the statistical section.

***Duration of participation***

The duration of participation of each subject will be at least 1 week to 3 months maximum (or 12 triplets).

**5- Design and conduct of research**

***Follow-up***

Each dialysis session includes a medical examination and a nurse monitoring. The information session is recorded in the case report form, some data are automatically transmitted by the generator to the dialysis Sined Medware ® software and recorded in the secure medical file of the patient. The estimated dialysability urea by the generator induces no extra cost.

Information contained in the protocol specification

At the beginning of the session

• weight, blood pressure, heart rate

• since the last session : weight gain and symptoms occurred

Monitoring of the dialysis session

• Continuous (Sined Medware ® Software generator Hospal Integra ®: blood flow, venous pressure, ultrafiltration rate, urea dialysance)

• every 30 ': blood pressure, heart rate.

• incidents during the session: clotting of the circuit (partial / full), clinical symptoms.

At the end of the session: session duration, weight, total urea dialysance

***Total duration of the Essay***

Inclusion (24 months months) and follow-up (1 week to 3 months): 27 months maximum

***Study exit***

The inclusion of a patient in the research protocol will end

• in case of premature termination (before three successive sessions are conducted)

• at the end of the maximum period of scheduled follow-up (3 months)

• withdrawal of patient’s consent (at any time)

• in case of permanent change of dialysis center

• in case of death

• in case of a technical superiority in the interim statistical analysis (see page 14)

Patients will be supported by the usual care practices in each dialysis center.

**6- Procedures evaluated**

**For all patients :**

- 3 hemodialysis sessions of 4 hours per week

- vascular access : arteriovenous fistula (mono or biponction with 15 G needles) or dual lumen central catheter

- blood flow : 250 to 300 ml / min

- dialysate : flow 500 ml / min, sodium conductivity 14.4 mS / cm ; bicarbonate conductivity 3.3 mS / cm, temperature 36 °C

- Dialysis membrane : AN 69 ST (NEPHRAL 400 ST)

**The procedures evaluated are:**

- Discontinuous rinses : injection of 125 ml of isotonic saline every 30 minutes on the arterial line

- Continuous Flushing (" dilution ") : continuous infusion of isotonic saline at a rate of 250 ml / h on arterial line during the dialysis session

- Adsorbed heparin: rinsing the dialysis membrane before the session with 2 l of isotonic saline containing 10,000 units of unfractionated heparin, no injection or infusion of isotonic saline during the dialysis session

**7- Endpoints**

***Main criterion***

Need to suspend the session for clotting of the dialysis circuit, because of either :

o complete coagulation of the circuit

o partial coagulation of the circuit (a clot is found in the lines or the bubble trap *)

o increased venous pressure (PV) of the circuit more than 50% beyond its initial value

* Note: the opacity of the fibers of the membrane NEPHRAL 400 ST makes it impossible the assessment of a partial internal filter clotting.

***Secondary endpoints***

1. Loss of dialysis efficiency

- Urea dialysance during the session (online measurement)
- hyperhydratation (weight at the end of the session: + 0,500 kg over the dry weight)

1. Dialysis session tolerance

- clinical secondary effects (headache, cramps, symptomatic arterial hypotension)
- any bleeding in the 48 heures following the dialysis session

**8- Data management and statistics**

***Justification of the number of patients to include***

The calculation of the number of subjects to include derives from the MacNemar test comparing groupes paired for a binary criteria:

|  | Response to treatment B | | | |
| --- | --- | --- | --- | --- |
| Response to the traitement of reference | Failure | Success | Total | Anticipated  Proportion |
| Failure | *r* | *s* | *r+s* | 1 - $\pi_{A}$ |
| Success | *t* | *u* | *t+u* | $\pi_{A}$ |
| Total | *r+t* | *s+t* | N_paires_ |  |
| Anticipated  Proportion | 1 - $\pi_{B}$ | $\pi_{B}$ |  |  |

This calculation is based on the estimation that $\psi$=s/t measures the degree by which a patient will have a success with the reference treatement and a failure with treatment B, compared to failure with the reference treatement and success with treatment B. Of note, patients (*r+u*) having success-success or failure- failure are not taking into account in this calculation. The latter is based on anticipation of marginal probabilities of success, $\pi_{A}$ et $\pi_{B}$, defined by the absence of circuit coagulation with each treatement:

- null hypothesis $\pi_{A}$ : 70% in the reference treatment group (30% of clotting), group A,
- $\pi_{B}$ = 80% in the dilution group (20% of clotting), groupe B,
- $\pi_{C}$ = 90% int the AN 69 ST membrane (10% of clotting), group C,

thus estimating the proportion of discordant pairs :$\pi$_discordant_ = (*s+t*)/N_pairs_ as well as $\psi$values

Accoring to published tables, sample size tables for clinical studies, Blackwell Science, 1997 :

- number of pais $N_{paires}=\frac{\left\{z_{1-\alpha/2}(\psi+1)+z_{1-\beta}\sqrt{\left[\left(\psi+1\right)^{2}-\left(\psi-1\right)^{2}\pi_{discordant}\right]}\right\}^{2}}{\left(\psi-1\right)^{2}\pi_{discordant}}$
- to compare A and B with alpha=5% an a power of at least 80 %, values are for $\pi$_discordant_ = 0.38 et $\psi$ = 1,7, i.e 294 pairs of dialysis sessions.
- to compare A and C with alpha=5% and a power of 80 %, values are for$\pi$_discordant_ = 0.34 et $\psi$ = 3,85, i.e 65 pairs of dialysis sessions.
- In sum, at least 294 triplets of dialysis sessions are needed, i.e. 882 dialysis sessions, corresponding to 65 patients to recruit over 12 months.
- Each patient being a cluster of dialysis sessions patient, the number of dialysis sessions has to be increased according ta an inflation coefficient based on putative intra-class correlation ( 0.001 to 0.01) ; the inflation coefficient is estimated to range from 1,002 to 1,5 for a mean cluster of 6 dialysis sessions, i.e a raise from 884 sessions to 1323. We finally estimate the need to perform 1050 dialysis sessions in 75 different patients.

***Analysis of collected data***

**Descriptive analysis**

Qualitatives data will be described in frequency and percentage, quantitative data by mean (±SD)-type) if distribution is normal, or median (quartiles) in other cases.

**Main Analysis**

For the main criteria, 2 analysis will be performed:

- At a treatment group level
- At an individual level

Approximately 60% of included patients will have a temporary contra-indication to systemic heparinisation ranging from 3 to 6 sessions (1 to 2 weeks). These patients will not be analysed individually and will contribute to the global principal analysis.

The global principal analysis will be performed according to 2 approches (MG Kenward and B Jones, The analysis of binary and categorical data from crossover trials, Statistical Methods in Medical Research, 1994 :325-344) :

- a marginal model estimating the effet of treatments taking into account other covariates (order effect or group effect, the Generalized Estimating Equations)
- a subject-specific model using a latent variable taking into account independent responses for a given subject réponses indépendantes pour un sujet. This model is also suited to low numbers of patients.

In the putatively 40% of patients (25 to 30 patients) that may have a prolonged contra-indication to heparin, anb individualized analysis will be performed (after at least 18 sessions to warrant sufficient power). Below 18 sessions, patients will only contribute to the global analysis, as described above.

For the individual analysis, The percentage of technical failures (technique A vs B, and also technique A vs C) will be performed. The exact Fisher test in this setting may be viewed as a randomisation test (ES Edington, Statistics and single case analysis, Progress in behavior modification. Academic Press, Inc. 1984;vol 16 :83-119). Appairment will not be taken into account. This is possible since the dialysis sessions and the efferent judgement criteria are statitically independent. Significativity threshold will be of 10% for a bilateral test, justified by the impoortance given to clinical significance (ES Edington).

At the end of this analysis:

- if one or several techniques are efficient, the patient will end the study
- if no technique apperas superior, the patient may continue the study for at least one triplet of sessions.

To avoid multiple analysis and alpha risk, a new analysis will be performed at 12 weeks, or at the end of the period of contra-indication to heparin. The alpha risk will be of 5% bilaterally.

Concerning the analysis of secondary endpoints, the same approach as above will be used for categorical variables. For quantitative variables, we also deem a treatment effect, period and order using a mixed model in which the subject will be returned as a random effect and to take into account the repeated nature of the data.

***Stopping rules***

If three consecutive failures to the same technique, randomization will continue on the remaining 2 techniques if the patient needs to continue its sessions without systemic heparin and if 3 months of inclusion are not met.

***Responsible for the data analysis (CNIL if necessary) and software.***

Statistical analysis will be carried out under the responsibility of Dr. Corinne Alberti, Coordinator of the Clinical Epidemiology Unit (CIE 5) of the Robert Debré Hospital. The software will be used SAS v 9.1 (Cary system) and S-plus (Mathsoft) for statistical data management.

**9- Management of serious adverse events, and monitoring of side effects**

#####

**Definitions :**

**Adverse events**

Any noxious and unintended event occurring in a person during a search, whether or not considered to be related to this

**Side effect**

Noxious and unintended response to an experimental treatment: medication (regardless of dose), device, surgical treatment ...) used in humans.

**Effect or serious adverse events**

Effect or adverse event that resultedin :

- Death

- A life-threatening situation

- A significant or sustainable disability or incapacity,

- A hospitalization or prolongation of hospitalization

- An anomaly or birth defect

- Other: any adverse effects deemed serious by the health professional, especially events requiring intervention to prevent one of the consequences noted above, some results of diagnostic tests.

**Unexpected adverse**

Adverse reaction, the nature, severity or evolution does not correspond to the information contained in the summary of product characteristics, the investigator's brochure or other repository recognized by the authorities.

**Accountability**

The relationship of intercurrent event with study drug is a relationship

1. direct

2. Possible: no direct relationship has been demonstrated, but its existence is possible or probable

3. No relationship: intercurrent event is not attributable to study medication

**Obligations of investigators:**

Non-serious adverse events

Not serious according to the above definition - any adverse events observed during the research and its results will be reported in the CRF in the section provided for this purpose.

One event must be reported by item. The event can be a symptom, a diagnosis or a supplementary examination results considered significant. All clinical or paraclinical evidence to best describe the corresponding event should be reported.

Any patient with an adverse event must be followed until resolution or stabilization of the latter, and changes will be noted on the page.

Serious adverse events

Investigators must inform in real time , the AP- HP- sponsor of any serious adverse events as defined above.

The investigator sends copies of serious adverse events sheets deviation of the CRF research, describing the EIG , by fax to DRRC on behalf of the project manager in charge of research 01 44 84 17 99 within 48 hours (if possible after an immediate call 01 44 84 17 23 in the event of unexpected death or life-threatening).

The investigator must for every serious adverse event issue a medical opinion on the possible relationship between the occurrence of the event and the protocol.

The clinical findings, diagnostic tests and appropriate laboratory tests be initiated to identify the origin of the reaction and the results of these investigations and the clinical course are reported.

Any new developments in research or in the context of research from literature data or current research must also be quickly notified to the sponsor.

**Reporting of serious adverse events to health authorities:**

***Adverse events expected***

Two main types of adverse events are expected:

Those related to the condition that led to the prescription of hemodialysis without systemic heparinization

The consequences of the clotting of the hemodialysis

**10 - Legal and Ethical Aspects**

**Role of promoter**

It is defined by the law 2004-806 of August 9, 2004. In this research, AP-HP is the promoter and the Regional Delegation for Clinical Research that provides the regulatory tasks will have a decisive role.

**Submission of protocol to CCPPRB**

In accordance with Article L.1123-6 of the Code of Health, the research protocol will be submitted to an advisory committee on the Protection of Persons in Biomedical Research of the Ile de France, with the agreement of the promoter (with certificate insurance and the receipt of fixed fee). The opinion of the Committee shall be notified in the form sent to the competent authority by the developer before the start of the search authority.

**CNIL**

The law provides that the statement must have been made before the actual start of the research.

The DRRC as a proponent will CNIL, in relation to the head of the computer file, at its annual statement simplified if the research is subject to a quality control data by CRA and within the scope application of the simplified procedure CNIL.

**Documentation of research**

Before starting the research, coordinating investigator provide the representative research sponsor a copy of his resume staff dated and signed with its registration with the College of Physicians number , as all investigators .

The protocol version accepted before submission with its annexes will be jointly signed by the investigator and coordinator representing the organization. If necessary, the scientist in charge will also be signed.

When a new version of the protocol, made necessary by amendments and / or governmental request, a new number and date will be awarded and the same signatures.

Each investigator will undertake to fulfill the obligations of the law and to conduct research in PCBs and respecting the terms of the Declaration of Helsinki. To do this, a signed and dated copy of the scientific commitment (document DRRC deviation) by each investigator for each participant in a clinical service center will be provided to the proponent.

**Patient consent**

Written informed consent of all patients must be obtained by the investigator before starting the protocol -specific procedures, in accordance with the principles of the current edition of the Declaration of Helsinki and legislation. Information will be given orally and summarized in a written document deemed appropriate by the ethics committee.

Patients, family members should have the opportunity to learn about the details of the study. The newsletter to the patient as well as the form of obtaining consent, made ​​by the investigator must have been approved by the ethics committee .

The consent form must be written in language understandable to the patient. Informed consent must contain the elements described in the Declaration of Helsinki and meet the legislation. The patient should read this document and the investigator must give the patient the opportunity to read it before signing it.

The consent must be signed by the patient and the more it will date itself consent. The signature confirms that consent is based on information that was included. Each patient must sign a consent form. Each original consent obtained will be filed and maintained by the investigator for possible inspection by regulatory authorities and / or in case of audit conducted by or on behalf of the coordinator.

**Quality Control and Quality Assurance**

The research will be framed according to the standard operating procedures of the AP-HP promoter.

The conduct of research in the study centers and support issues will be made ​​in accordance with the Declaration of Helsinki and Good Practices.

*Monitoring procedures*

The sponsor representatives will visit the study centers at a rate corresponding to the diagram of monitoring patients in the protocol, the inclusions in the various centers and the level of risk that has been assigned to the protocol.

First, before inclusions, for opening each center with the implementation of the protocol and getting to know the investigators.

On subsequent visits, case report forms will be reviewed as and when the progress of research by CRAs representing the developer who will control the filling and ensure good data validation. The principal investigator of each center and other investigators that include or follow the subjects participating in the research agree to receive sponsor representatives appointed by the AP- HP at regular intervals.

During these site visits and in accordance with Good Clinical Practice, the following will be reviewed :

- Compliance with the research protocol and procedures that are defined

- Examination of the source and confrontation with the instructions given in the case report form data documents

- Quality assurance of data in the notebook of observation accuracy, missing data, data consistency, according to the rules laid down by the procedures DRRC

**Transcription of data in the CRF**

All information required by the protocol must be provided in the case report form and given by the investigator for each missing explanation.

The data will be transferred in the case report forms as and when they are obtained whether it is clinical or para- clinical data must be copied clearly and legibly in black ink these books (in order to facilitate the duplication and data entry).

Erroneous data tracked on case report forms will be clearly crossed out and the new data will be copied to the notebook with the initials and date by the member of the team of investigators who have made ​​the correction.

The anonymity of the subjects will be provided by reference to the maximum of the first three letters of the name and the first two letters of the name of the subject on all documents necessary for research, or deletion by appropriate means (white checker ...) data registered on the copies of source documents for the documentation of research.

Computerized data to a file will be declared to the CNIL under the procedure adapted to the case.

**Amendments to the research protocol**

The DRRC must be informed of any proposed changes to the protocol by the coordinating investigator. The changes will be described in substantial or not.

Any amendments to the research protocol, the ethics committee must be notified if it causes substantial changes, that is to say if the planned changes are likely, one way or another, change the guarantees provided people who are suitable for biomedical Research (changing an inclusion criterion, extended for a period of inclusion, participation of new investigators .....).

**Extension of the Research**

Any extension of the research (deep change of regimen or populations included, or extension of treatment and therapeutic procedures not originally planned in the protocol) should be considered as a new search.

**Responsibility**

The Assistance Publique-Hôpitaux de Paris is the sponsor of the research. In accordance with the law on biomedical research, she took out insurance with the company Gerling KONZERN for the duration of the research, ensuring its own liability as well as any stakeholder (doctors or staff involved in the implementation of the research) (Act No. 2004-806, Art L.1121-10 of the CSP).

The Assistance Publique - Hôpitaux de Paris reserves the right to interrupt the search at any time for medical or administrative reasons, in this case, a notification will be provided to the investigator.

**Final research report**

The final research report will be written in collaboration with the coordinator and biostatistician for this search. This report will be submitted to each of the investigators for review . Once a consensus has been reached, the final version must be endorsed by the signature of each of the investigators and sent to the developer as soon as possible after the effective end of the search . A report in the reference plane of the competent authority must be submitted to the competent authority and the Committee within one year after the end of the research, being understood as the last follow-up visit last topic included. This period is reported at 90 days in case of premature termination of the research .

**Publications and data properties**

AP-HP owns the data and no use or transmission to a third party may be made without prior approval.

First be signed publications, people actually engaged in protocol development and progress as well as the drafting of results.

The Assistance Publique -Hôpitaux de Paris should be mentioned as the sponsor of biomedical research and financial support as appropriate. The terms " Assistance Publique -Hôpitaux de Paris " should appear in the address of the authors.

**APPENDIX : Bibliography**

Apsner, R., H. Buchmayer, et al. (2001). "Simplified citrate anticoagulation for high-flux hemodialysis." Am J Kidney Dis **38**(5): 979-87.

- Bartels, P. C., M. Schoorl, et al. (2000). "Activation of coagulation during treatment with haemodialysis." Scand J Clin Lab Invest **60**(4): 283-90.
- Bouffard, Y., B. Delafosse, et al. (1989). "[Low molecular weight heparins in hemodialysis. Value in patients with acute renal insufficiency at high risk of hemorrhage]." Presse Med **18**(14): 726-7.
- Camici, M., L. Evangelisti, et al. (1997). "Coagulation activation in extracorporeal hemodialysis." Int J Artif Organs **20**(3): 163-5.
- Canivet, E., A. Wuillai, et al. (2003). "Systemic heparin-free hemodialysis : how to treat patients at risk of bleeding." J Am Soc Nephrol **14**: A728.
- Caruana, R. J., R. M. Raja, et al. (1987). "Heparin free dialysis: comparative data and results in high risk patients." Kidney Int **31**(6): 1351-5.
- Delawari, J., J. Renaux, et al. (2001). "Benefits of AN69ST membrane on heparin-free dialysis." Nephrol Dial Transplant **16**(6): A174.
- Flanigan, M. J., J. Von Brecht, et al. (1987). "Reducing the hemorrhagic complications of hemodialysis: a controlled comparison of low-dose heparin and citrate anticoagulation." Am J Kidney Dis **9**(2): 147-53.
- Hampers, C. L., M. D. Balufox, et al. (1966). "Anticoagulation rebound after hemodialysis." N Engl J Med **275**(14): 776-8.
- Janssen, M. J., P. C. Huijgens, et al. (1993). "Citrate versus heparin anticoagulation in chronic haemodialysis patients." Nephrol Dial Transplant **8**(11): 1228-33.
- Kelleher, S. P. and G. Schulman (1987). "Severe metabolic alkalosis complicating regional citrate hemodialysis." Am J Kidney Dis **9**(3): 235-6.
- Keller, F., J. Seemann, et al. (1990). "Risk factors of system clotting in heparin-free haemodialysis." Nephrol Dial Transplant **5**(9): 802-7.
- Lavaud, S., E. Canivet, et al. (2002). "La fixation d'héparine sur la membrane AN 69 n'altère pas ses propriétés anticoagulantes." Néphrologie **23**(6): 288.
- Lavaud, S., E. Canivet, et al. (2003). "Optimal anticoagulation strategy in haemodialysis with heparin-coated polyacrylonitrile membrane." Nephrol Dial Transplant **18**(10): 2097-104.
- Lindholm, D. D. and J. S. Murray (1964). "A simplified method of regional heparinization during hemodialysis according to a predetermined dosage formula." Trans Am Soc Artif Intern Organs **10**: 92-7.
- Martin-Malo, A., F. Velasco, et al. (1993). "Fibrinolytic activity during hemodialysis: a biocompatibility-related phenomenon." Kidney Int Suppl **41**: S213-6.
- Nurmohamed, M. T., J. ten Cate, et al. (1991). "Long-term efficacy and safety of a low molecular weight heparin in chronic hemodialysis patients. A comparison with standard heparin." ASAIO Trans **37**(3): M459-61.
- Pinnick, R. V., T. B. Wiegmann, et al. (1983). "Regional citrate anticoagulation for hemodialysis in the patient at high risk for bleeding." N Engl J Med **308**(5): 258-61.
- Preuschof, L., F. Keller, et al. (1988). "Heparin-free hemodialysis with prophylactic change of dialyser and blood lines." Int J Artif Organs **11**(4): 255-8.
- Romao, J. E., Jr., M. A. Fadil, et al. (1997). "Haemodialysis without anticoagulant: haemostasis parameters, fibrinogen kinetic, and dialysis efficiency." Nephrol Dial Transplant **12**(1): 106-10.
- Schultze, G., S. Hollmann, et al. (1992). "Formation of thrombin-antithrombin III complex using polyamide and hemophan dialyzers." Int J Artif Organs **15**(6): 370-3.
- Sepulveda, S., L. Davis, et al. (1997). "Blood transfusion during heparin-free hemodialysis." Kidney Int **51**(6): 2018-21.
- Sperschneider, H., R. Deppisch, et al. (1997). "Impact of membrane choice and blood flow pattern on coagulation and heparin requirement--potential consequences on lipid concentrations." Nephrol Dial Transplant **12**(12): 2638-46.
- Swartz, R. D. (1981). "Hemorrhage during high-risk hemodialysis using controlled heparinization." Nephron **28**(2): 65-9.
- Swartz, R. D. and F. K. Port (1979). "Preventing hemorrhage in high-risk hemodialysis: regional versus low-dose heparin." Kidney Int **16**(4): 513-8.
- Tielemans, C., C. Neyens, et al. (2001). "Hémodialyse sans anticoagulation systémique grâce à la membrane AN69ST." Néphrologie **22**(5): 263.
- Weitz, J. I. (1997). "Low-molecular-weight heparins." N Engl J Med **337**(10): 688-98.

**Information and Consent Form**

**for a person participating in biomedical research**

Chronic hemodialysis without systemic heparinization

Dr. ................. (1 ), physician investigator asked me to participate in biomedical research entitled " chronic hemodialysis without systemic heparinization ."

Assistance Publique -Hôpitaux de Paris, the promoter of this assay has insurance in accordance with the law. The doctor said that I was free to accept or refuse to participate in this research.

To inform my decision, I have received and understood the following information:

Hemodialysis typically involves the injection of an anticoagulant (heparin-like) to prevent clotting of blood in the dialysis circuit. However, because of my current situation, the use of heparin is against inappropriate to temporarily or permanently. This situation is not unique because it accounts for about 10 % of dialysis sessions performed at Necker Hospital.

When systemic heparin - against is shown, three different methods may be used to prevent coagulation of blood in the dialysis circuit. These methods are effective in the majority of cases . This is either ( i ) flushing the system with a 125 ml of isotonic saline ( 9 ‰ NaCl ) every 30 minutes , or ( ii ) continuous infusion of 250 ml / hour of isotonic saline in the circuit or ( iii) the use of a special membrane dialysis, NEPHRAL 400 ST with adsorbed heparin. These three methods are currently used in the dialysis center of the Necker Hospital, and the technical choice is left open to the discretion of the physician when the session begins. There is no rational criterion of "best choice" because no statistical analysis is not currently possible to say which of the three methods is the most effective .

To answer this question, a comparative test is useful. This essay is currently underway at the Necker Hospital , and provides a total participation of 78 different patients. When a patient is included in the test , it is treated by three methods as the use of systemic heparin is contra-indicated in his case (or at least one week to six months) . It is a randomization at the time of inclusion in the study that lets you know the order in which the techniques are used throughout the dialysis sessions of the week: for example, there may be technique (iii ) on Monday and ( ii ) on Wednesday and finally ( i) on Friday .

Statistical analysis performed at study will tell if, for example, the technique iii or not to reduce the frequency of coagulation of the dialysis circuit and whether it should therefore be favored in the future : it s' is the main objective of the study . Another goal , said secondary, is to determine know technique provides the best removal of uremic toxins during the dialysis session .

For patients participating in the study, there is no particular foreseeable specific risks. Risks for patients to whom this study is proposed are those related to the situation contra - indicating the use of systemic heparin, and possible consequences of the loss of blood clotting of the dialysis circuit .

This research received the favorable opinion of the Advisory Committee on Protection of Persons participating in Biomedical Research Paris Necker

***Published by OJ of 19 February 2006 decision of the CNIL January 5, 2006***

"In the context of biomedical research in which the Assistance Publique - Hôpitaux de Paris invites you to participate in a treatment of your personal data will be used to help analyze the results of research in light of the objective the latter has been presented to you.

To this end, the medical information about you data on your lifestyle will be forwarded to the sponsor of the research or persons or companies acting on its behalf. These data will be identified the first three letters of your name. These data may also, under conditions ensuring their confidentiality, be communicated to the French health authorities, other bodies of the Assistance Publique - Hôpitaux de Paris. Accordance with the provisions of the law relating to data files and freedom, you have a right of access and rectification. You also have the right to object to the transmission of data covered by professional secrecy may be used in this research and to be treated.

You can also access directly or through a doctor of your choice to all of your medical information under the provisions of Article L. 1111-7 Code of Public Health .

These rights shall be exercised by the physician who follows you through the research and who knows your identity. "

After discussing and getting answers to all my questions, I freely accept and voluntarily participate in the research described above. I am fully aware that I can withdraw my consent at any time to my participation in this research and that whatever my reasons and without liability. Failure to participate in such research does not affect my relationship with the physician investigator who offer me, if I want and if necessary, another treatment.

I can at any time request any additional information or to Drs Joly Dr. GUERY (No. telephone 01 44 49 54 56).

If I wish, to an end, I will be informed by the investigator who collects my consent overall results of this research.

My consent discharge anything the investigator and the sponsor of all their responsibilities and I keep all my rights guaranteed by law.

Done in PARIS, (date)

The investigator : Person giving consent :

Full name: Full name:

Signature Signature
